# Repeated cold exposures protect a mouse model of Alzheimer's disease against cold-induced tau phosphorylation

**DOI:** 10.1016/j.molmet.2019.01.008

**Published:** 2019-01-26

**Authors:** Marine Tournissac, Philippe Bourassa, Ruben D. Martinez-Cano, Tra-My Vu, Sébastien S. Hébert, Emmanuel Planel, Frédéric Calon

**Affiliations:** 1 Faculty of Pharmacy, Université Laval, Quebec City, Qc, Canada; 2 Neurosciences Axis, CHU de Québec-Université Laval Research Center, Quebec City, Qc, Canada; 3 Department of Psychiatry and Neuroscience, Faculty of Medicine, Université Laval, Quebec City, Qc, Canada

**Keywords:** Alzheimer's disease, brown adipose tissue, tau phosphorylation, thermoregulation, 3xTg-AD mice

## Abstract

**Objective:**

Old age is associated with a rise in the incidence of Alzheimer's disease (AD) but also with thermoregulatory deficits. Indicative of a link between the two, hypothermia induces tau hyperphosphorylation. The 3xTg-AD mouse model not only develops tau and amyloid pathologies in the brain but also metabolic and thermoregulatory deficits. Brown adipose tissue (BAT) is the main thermogenic driver in mammals, and its stimulation counteracts metabolic deficits in rodents and humans. We thus investigated whether BAT stimulation impedes AD neuropathology.

**Methods:**

15-month-old 3xTg-AD mice were subjected to repeated short cold exposures (RSCE), consisting of 4-hour sessions of cold exposure (4 °C), five times per week for four weeks, compared to animals kept at housing temperature.

**Results:**

First, we confirmed that 3xTg-AD RSCE-trained mice exhibited BAT thermogenesis and improved glucose tolerance. RSCE-trained mice were completely resistant to tau hyperphosphorylation in the hippocampus induced by a 24-hour cold challenge. Finally, RSCE increased plasma levels of fibroblast growth factor 21 (FGF21), a batokine, which inversely correlated with hippocampal tau phosphorylation.

**Conclusions:**

Overall, BAT stimulation through RSCE improved metabolic deficits and completely blocked cold-induced tau hyperphosphorylation in the 3xTg-AD mouse model of AD neuropathology. These results suggest that improving thermogenesis could exert a therapeutic effect in AD.

## Highlights

• Cold acclimation increases brown adipose tissue thermogenesis in old 3xTg-AD mice.
• Cold acclimation improved glucose tolerance in old 3xTg-AD mice.
• Enhanced thermogenesis protects against cold-induced brain tau phosphorylation.
• Repeated cold exposures increased plasmatic levels of fibroblast growth factor 21.
• Peripheral fibroblast growth factor 21 levels correlate with tau phosphorylation.

## 1 Introduction

Alzheimer's disease (AD) is a neurodegenerative disease characterized by disrupted cognitive functions and is diagnosed neuropathologically by the presence of tau-laden neurofibrillary tangles and Aβ-loaded plaques [1], [2]. The principal risk factor for developing AD is unquestionably old age [3]. Therefore, a better understanding of pathological changes triggered by old age could reveal new clues in AD pathogenesis. Among the changes that come with aging, thermoregulatory deficits are frequently reported. Indeed, elderly individuals regulate their core temperature less efficiently than younger adults [4], and many of them have subnormal body temperatures during the day [5], [6], along with decreased basal metabolism [7]. Interestingly, the appearance of defects in maintaining optimal body temperature roughly coincides with a rise in the incidence of AD in this population.

Animal studies are providing new lines of evidence suggesting that thermoregulatory deficits contribute to AD pathogenesis. It has been repeatedly shown in the rodent brain that a decrease in body temperature induces tau hyperphosphorylation [8], [9], [10], [11], [12], one of the main neuropathological markers of the disease [1], [2]. Our studies in animal models show that both aging and AD pathology potentiate the rise in tau phosphorylation following acute exposure to cold (4 °C, 24 h) [11], [12]. For instance, compared to non-transgenic mice, the 3xTg-AD mouse model of AD has a lower basal body temperature that worsens with age and the progression of the neuropathology. Interestingly, cold exposure was shown to aggravate AD neuropathology, while thermoneutrality (28 °C) improved both AD-related neuropathologies and cognitive impairment [11], [12].

Thermoregulatory deficits can also be interpreted as part of the general metabolic failure that is believed to occur in AD. Strong pathogenic links between AD and diabetes have been revealed in numerous reports in recent years, as type 2 diabetes is now considered a risk factor for developing AD [13], [14]. Reciprocally, AD patients display lower brain glucose metabolism and impaired insulin signaling [15], [16]. In addition, peripheral metabolic deficits have been observed in animal models expressing AD neuropathology [17], [18]. Interestingly, mouse models of diabetes and obesity also display thermoregulatory deficits associated with higher tau phosphorylation, further supporting a close relationship between type 2 diabetes and AD [19], [20]. Not surprisingly, numerous antidiabetic drugs have been tested for AD in preclinical and clinical studies, including insulin, thiazolidinediones, metformin, and GLP-1 analogs [21], [22].

One promising strategy for simultaneously correcting thermoregulatory and metabolic deficits is through the stimulation of brown adipose tissue (BAT) thermogenesis. BAT is the main thermogenic tissue in mammals. It burns fat and glucose to produce heat that dissipates throughout the body via blood flow [23]. Hence, the activation of BAT has been considered as a potential drug target for type 2 diabetes and obesity for a long time [24], with renewed interest following the demonstration that (i) BAT is functional in adults [25] and (ii) BAT activity can be modulated through cold exposure or drugs [26]. In addition, active BAT has the remarkable ability to improve glucose tolerance and insulin sensitivity [27], [28]. Since older individuals experience a decrease in BAT activity [29], they are likely to benefit the most from BAT stimulation.

BAT is not only a modulator of energy homeostasis but also an endocrine organ that secretes metabolic regulators or adipokines, also called batokines, such as fibroblast growth factor 21 (FGF21), adiponectin, and interleukin 6 (IL-6) [30]. Interestingly, FGF21 was shown to reverse high-fat diet-induced cognitive defects in rats [31], reduce blood–brain barrier disruptions in db/db mice [32], act as a neuroprotective agent against glutamate-induced excitotoxicity in primary rat brain neurons [33], [34], and protect against Aβ42 toxicity in vitro [35].

To our knowledge, no study has investigated the stimulation of BAT thermogenesis in the context of AD. Given that BAT is involved in multiple pathways in AD pathogenesis, we hypothesized that correcting thermoregulatory impairments through BAT stimulation could exert benefits on metabolic and neuropathological endpoints in a mouse model of AD. Repeated short cold exposures (RSCE) is a standardized procedure to stimulate BAT growth and activity that has been shown to increase energy expenditure and improve glucose tolerance and insulin sensitivity in rodents [36], [37], [38] and humans [39], [40], [41].

We thus applied RSCE to a mouse model of AD (the 3xTg-AD mouse) that reproduces both metabolic and thermoregulatory deficits along with the development of tau and amyloid pathologies in the brain [12], [18], [21], [42]. Our objective was to determine whether stimulating BAT thermogenesis could be a potential therapeutic strategy in AD.

## 2 Methods

### 2.1 Animals and diet

Thirteen-month-old 3xTg-AD (APP_swe_, PS1_M146V_, tau_P301L_) mice produced at our animal facility were used in equal numbers of males and females in each group. Mice from our colony were fed the same chow (Teklad 2018, Harlan Laboratories, Canada) from breeding to 13 months of age. From 13 to 16 months, mice were fed a 20% (w/w) of total fat diet to be more representative of a human “Western diet” and to potentiate metabolic defects previously observed in old 3xTg-AD mice [18]. The diet was manufactured by Research Diets Inc. (New Brunswick, NJ) and is described in detail in Table S1. We used purified diet formulations standardized to ensure consistency and eliminate batch-to-batch variations, containing measured concentrations of macronutrients, vitamins, and minerals. All experiments were performed in accordance with the Canadian Council on Animal Care and were approved by the Institutional Committee at the Centre Hospitalier de l’Université Laval (CHUL).

### 2.2 Cold exposures

**Figure 1:**
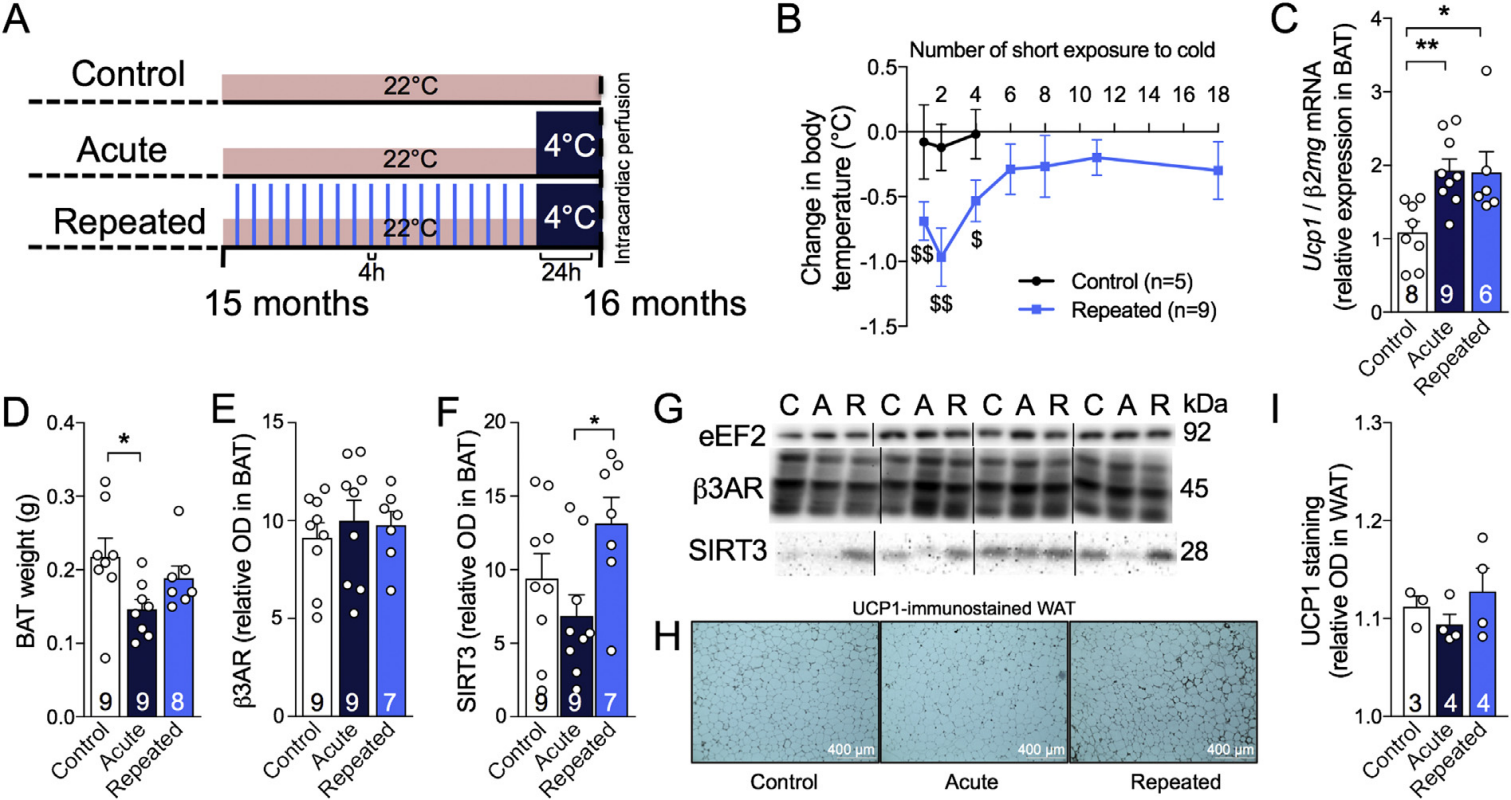
Repeated short cold exposures protect old 3xTg-AD mice against cold-induced decrease in body temperature by increasing thermogenesis. A: Study timeline, including RSCE. B: Difference in rectal temperatures of exposed and non-exposed mice taken before and after each short exposure to cold (4 h, 4 °C) between 15 and 16 months of age. The temperature of the mice was recorded before and after the first, the second, the 4th, the 6th, the 8th, the 13th and the 18th (last) repeated short cold exposure (RSCE) (4 °C during 4 h). C: BAT *Ucp1* mRNA expression normalized to *β2mg* (qPCR). D: BAT weighed just after dissection. E: Levels of β3AR and F: SIRT3 proteins measured in BAT by western blot. G: Examples of Western Blots. Homogenates were all run on the same gel, but consecutive bands were not taken for all representative photo examples. H: Immunohistochemistry of UCP1 on WAT sections. I: Quantification of UCP1 staining in WAT. Data are represented as mean ± SEM (n/group indicated in bars). Statistical analyses: One sample t-test versus 0: ^#^p < 0.05; ^##^p < 0.01. One-way ANOVA, Tukey's post-hoc test: *p < 0.05; **p < 0.01. Control (C): 22 °C; Acute (A): 24 h, 4 °C; Repeated (R): 4 h, 4 °C for four weeks + 24 h, 4 °C. Abbreviations: *β2mg*: β2 microglobulin; β3AR: β3 adrenergic receptor; BAT: brown adipose tissue; eEF2: eukaryotic elongation factor 2; Ucp1: uncoupling protein 1; SIRT3: Sirtuin 3; WAT: white adipose tissue.

From 15 to 16 months, all animals were housed one per cage to avoid mutual heating. A control group remained at housing temperature (22 °C) for the duration of the study (Figure 1A). The “Acute” group underwent a 24-hour period of exposure at 4 °C, without adaptation, just before sacrifice to assess thermoregulatory capacity. The “Repeated” group underwent RSCE consisting of 4-h exposure at 4 °C (from 10 a.m. to 2 p.m.), five days per week for four weeks. This RSCE protocol sums up to a total of 18 4-h sessions of short exposure to cold. Then, animals in the "Repeated" group underwent a final 24-hour exposure at 4 °C (from 9 a.m. to 9 a.m., identical to the ‘Acute’ group) at the end of the fourth week (Figure 1A) [36], [37], [38]. To minimize stress variability between the groups, all animals were moved from the housing room to the room next to the cold chamber. Body temperature was determined using an electronic thermometer coupled with a rectal probe before and after cold exposure sessions at the same time of the day. All mice were sacrificed by intracardiac perfusion at their respective temperatures (4 °C or 22 °C) as described previously [12]. Although it is known that anesthesia affects body temperature [43], all mice were put under deep anesthesia with ketamine/xylazine i.p. injection (100 mg/kg ketamine, 10 mg/kg xylazine) for ethical reasons a few minutes before intracardiac perfusion. Interscapular BAT, gastrocnemius muscles, and brains were rapidly dissected and frozen at −80 °C until processed.

### 2.3 Glucose tolerance test

Glucose tolerance test (GTT) was performed after the fifteenth short cold exposure session, or after three weeks of RSCE, during the few days without short cold exposure (Figure 1A). “Control” and “Acute” groups were pooled under the “Control” group because at the time of the GTT, “Acute” mice did not yet undergo the acute exposure to cold (24 h, 4 °C) and were still in the same conditions as the “Control” group (22 °C). In addition, it was not possible to test the mice after the final 24-h acute cold exposure because mice were immediately sacrificed at 4 °C. Sixteen-month-old 3xTg-AD non-exposed and RSCE mice (after the fifteenth exposure) were fasted for 6 h. Glucose (1 g/kg) was injected i.p. and blood glucose was measured over a 2-h period with a glucometer (OneTouch UltraMini; LifeScan, Milpitas, CA) using a blood drop sampled from the saphenous vein.

### 2.4 Protein extraction

For the parieto-temporal cortex, protein extraction method results in a TBS-soluble intracellular and extracellular fraction (cytosolic fraction), a detergent-soluble fraction (membrane fraction) and a detergent-insoluble fraction (resuspended in formic acid) as previously described [44]. For the hippocampus, frozen samples were directly homogenized in a lysis buffer containing detergents that results in a detergent-soluble fraction (cytosolic and membrane fraction). The pellets remaining from ultracentrifugation were resuspended in formic acid (detergent-insoluble fraction). Proteins from the BAT and gastrocnemius muscles were extracted in the lysis buffer only.

### 2.5 Western immunoblotting

The detailed method for western immunoblotting is described elsewhere [18]. The list of primary and secondary antibodies used in our experiments is available in Supplementary Table 4. Homogenates were all run on the same gel for each experiment. Proteins extracted from the hippocampus of tau KO mice were added in immunoblotting experiments with tau antibodies as a negative control, as described elsewhere [45].

### 2.6 Aβ40 and Aβ42 quantification

For β-Amyloid ELISA, protein extracts from the parieto-temporal cortex were used. Aβ40 and Aβ42 were measured in TBS-soluble and detergent-insoluble fractions using a human β-Amyloid ELISA (Wako, Osaka, Japan) according to the manufacturer's instructions [44]. Plates were read at 450 nm using a SynergyTM HT multidetection microplate reader (Biotek, Winooski, VT).

### 2.7 Quantitative real-time PCR

The expression levels of uncoupling protein 1 (*Ucp1,* TaqMan Gene Expression Assays, Mm01244861_m1, Life Technologies), *Ffg21* (TaqMan Gene Expression Assays, Mm00840165_g1, Life Technologies) and β2-microglobulin (*β2mg*, Mm00437762_m1, Life Technologies) mRNA were determined in BAT using a reverse transcription real-time quantitative PCR (RT-qPCR) as previously described [11]. The delta delta Ct (ddCT) method was used to evaluate differences in relative gene expression between groups using *β2mg* as the control gene. Results are presented as ratios of *Ucp1*/*β2mg* or *Fgf21*/*β2mg* cDNA relative to the control group.

### 2.8 FGF21 assay

Levels of FGF21 were determined in plasma sampled before intracardiac perfusion in non-fasted mice and in BAT protein extracts using the mouse/rat FGF21 Quantikine ELISA kit (MF2100, R&D systems, Minneapolis, MN).

### 2.9 Triglycerides assay

Levels of triglycerides were measured in plasma sampled before intracardiac perfusion in non-fasted mice using the Thermo Scientific Triglycerides Reagent assay (TR22421, Thermo Fisher Scientific, Waltham, MA, USA).

### 2.10 Histology and UCP1 immunostaining

Visceral (perirenal and epididymal) and subcutaneous (inguinal) fat pads were sampled and weighed. Epididymal fat was post-fixed for 48 h in 4% paraformaldehyde (pH 7.4). Samples were then transferred in PBS until embedding in paraffin. Samples were cut in 10-μm-thick slices and mounted. For adipocytes quantification, slices were stained in hematoxylin and eosin (H&E) as described elsewhere [21]. Adipocyte area and number were quantified using ImageJ software (version 1.50i, NIH, Bethesda, MA, USA). For UCP1 immunostaining, slices were first deparaffinized, followed by antigen retrieval with a boiling 10 mM sodium citrate buffer (pH 6.0) for 30 min and inhibition of endogenous peroxidases with a 3% hydrogen peroxide in methanol for 30 min. Mounted slices were then washed twice in PBS with 0.4% Triton X-100 (PBS-T) and 1% horse serum for 10 min before blocking with 5% horse serum in PBS-T solution for 30 min. Slices were incubated overnight at 4 °C in a humid chamber with a rabbit UCP1 antibody (1:200, Ab10983, Abcam, Cambridge, United Kingdom) in a 1% horse serum PBS-T solution. Sections were then washed twice in PBS-T for 10 min before a 1-h incubation period with a goat anti-rabbit biotinylated antibody (1:200, Jackson ImmunoResearch, PA, USA). Mounted sections were then washed twice in PBS-T and an avidin/horseradish peroxidase complex (ABC Elite Kit; Vector Laboratories, Burlington, ON, Canada) was added for 35 min following the manufacturer's guidelines. After two washes, a 0.3 mg/mL 3-amino-9-ethylcarbazole (AEC) (Sigma–Aldrich, St Louis, MO, USA) and 0.03% hydrogen peroxide in acetate buffer was added for detection. The reaction was stopped by extensive washings in PBS, and sections were then coverslipped with Mowiol mounting medium. Images were taken with an EVOS® FL Auto Cell Imaging System (Thermo Fisher Scientific, Waltham, MA, USA). Optical density was quantified with ImageJ software (NIH, version 1.50i).

### 2.11 Corticosterone ELISA

Corticosterone levels were measured in plasma sampled from the saphenous vein just before and after moving to the cold chamber in non-fasted mice following the manufacturer's instructions (Ab108821, Abcam, Cambridge, United Kingdom).

### 2.12 Statistical analysis

Data are represented as a mean ± standard error of the mean (SEM), except for Tables S2 and S3 in which data are presented as mean ± standard deviation (SD). The statistical analysis and the number of mice per group are specified in each figure. Bartlett's tests were used to rule out inequality of variances between groups. One-way (one independent variable) or two-way (two independent variables) ANOVAs were used when more than two groups were compared. ANOVAs were followed by a Tukey's post-hoc analysis in cases of equal variance. In cases of unequal variance, a Kruskal Wallis followed by a Dunn's post-hoc test were performed. Repeated measures two-way ANOVAs followed by a Bonferroni's post-test were executed to compare recurrent measurements in the same animals. An unpaired Student's t-test was performed when only two groups were compared. One sample t-test was used to compare means to a theoretical value. Correlations between variables were investigated using linear regression analyses. All statistical analyses were performed with Prism 5 (GraphPad software, San Diego, CA, USA) or JMP (version 12.1.0; SAS Institute Inc., Cary, IL, USA) software and statistical significance was set at p < 0.05.

## 3 Results

### 3.1 Repeated short cold exposures potentiate BAT thermogenic capacity and protect old 3xTg-AD mice against cold-induced decrease in body temperature

RSCE is a standardized procedure to stimulate BAT growth and thermogenesis activity [36], [37], [38]. Corticosterone levels were measured in plasma sampled before and after the first and the thirteenth RSCE to confirm that stress was equivalent between cold-exposed and non-exposed mice (Figure S1). We first observed that mice in the RSCE group became resistant to cold-induced decrease in body temperature from the sixth cold exposure session onward, as shown by a non-significant change in body temperature before and after short exposures (Figure 1B and Figure S2 for raw data). We confirmed that *Ucp1* mRNA expression in BAT was increased by cold exposure, after a single acute episode (24 h at 4 °C) or at the end of the RSCE session (Figure 1C), while β3 adrenergic receptors (β3AR) were not affected by RSCE (Figure 1E,G). BAT weights were reduced by acute cold exposure, possibly due to brown adipocyte shrinkage following lipid consumption to produce heat (Figure 1D). We also found an increase in SIRT3 in the BAT of repeatedly cold-exposed mice (Figure 1F,G), a protein expressed in mitochondria of BAT after fasting or cold exposure conditions [46], [47], [48], further evidencing that RSCE efficiently stimulated BAT thermogenesis. Since RSCE may improve whole-body thermogenesis not only through BAT stimulation, we assessed non-shivering activity in muscles through sarcolipin protein (Table S2) [49]. However, we did not observe any significant increase in muscular proteins involved in thermogenesis. Finally, a trend toward a “browning” phenotype was observed in the white adipose tissue (WAT) of repeatedly cold-exposed mice with UCP1 staining (Figure 1H), although the quantification of the optical density of UCP1 staining was not statistically different from control mice (Figure 1I).

### 3.2 Repeated short cold exposures improve glucose tolerance in 16-month-old 3xTg-AD mice

**Figure 2:**
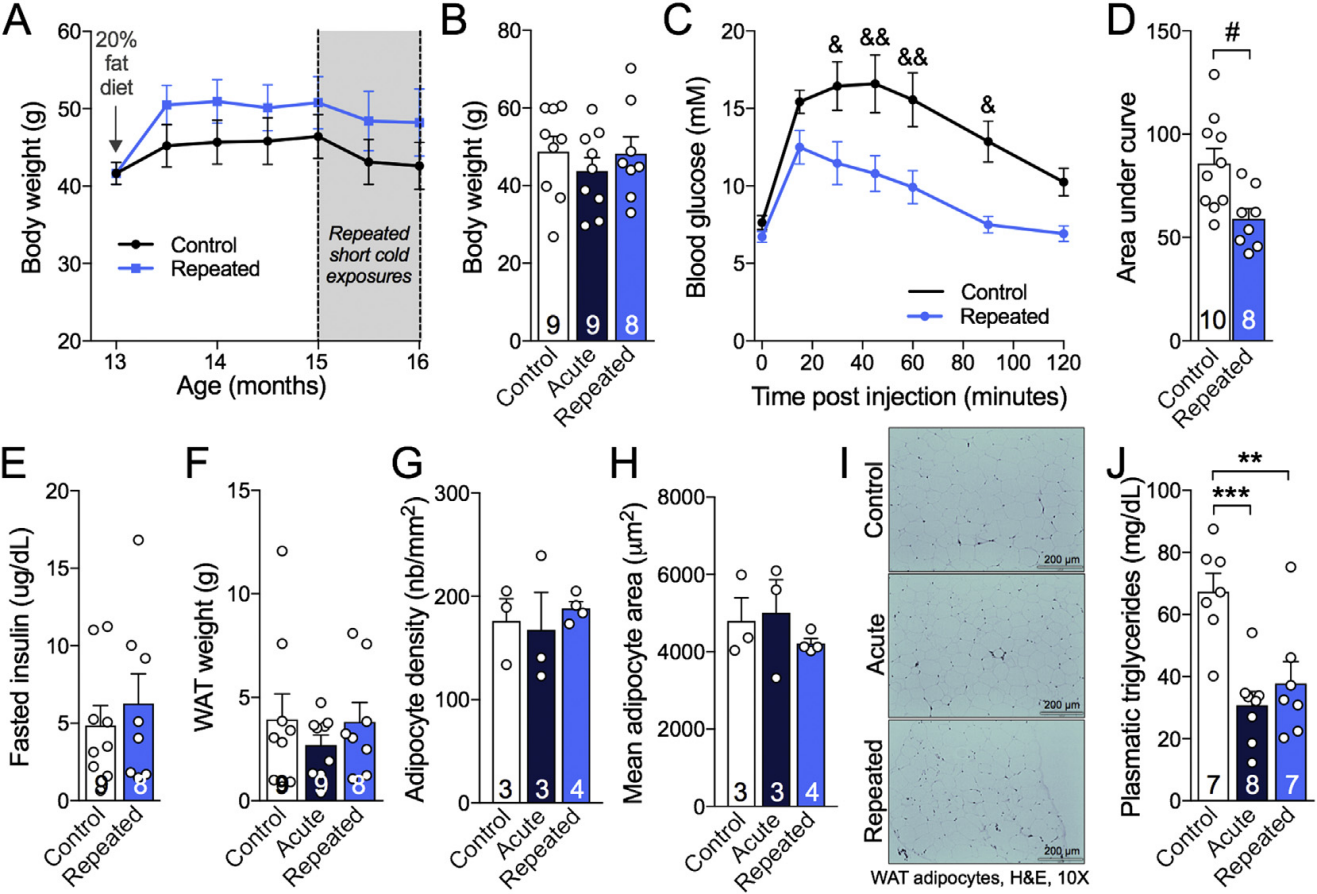
Repeated short cold exposures improve glucose tolerance in 16-month-old 3xTg-AD mice and decrease plasma triglycerides without affecting white adipose depots. A: Body weights during the dietary treatment and series of repeated short cold exposures. B: Body weights at the end of the experiment. C,D: Glucose tolerance test performed after the fifteenth short cold exposure session. E: Levels of insulin measured in plasma sampled after the 6-hour fasting (before GTT). F: Visceral (epididymal and perirenal fat) and subcutaneous (inguinal) adipose tissue of 16-month-old 3xTg-AD mice weighed just after dissection. G,H: Number and average surface of adipocytes in epididymal fat. I: Photos of white adipocytes (H&E stained paraffin sections). J: Levels of triglycerides measured in plasma sampled at the sacrifice in non-fasted mice. Data are represented as mean ± SEM (n/group indicated in bars). Statistical analyses: Repeated measures two-way ANOVA, Bonferroni's post-hoc test: ^&^p < 0.05; ^&&^p < 0.01. Unpaired Student t-test: ^#^p < 0.05. One-way ANOVA, Tukey's post-hoc test: **p < 0.01; ***p < 0.001. Control: 22 °C; Acute: 24 h, 4 °C; Repeated: 4 h, 4 °C for four weeks + 24 h, 4 °C. Note: “Control” and “Acute” groups were pooled under the “Control” group in A and C because at the time of the GTT and the weighing, “Acute” mice did not yet undergo the acute exposure to cold (24 h, 4 °C) and were still in the same conditions as the “Control” group (22 °C).

Increased BAT thermogenesis activity leads to improved metabolic outcomes, including glucose tolerance in rodents [38]. We took advantage of the fact that 3xTg-AD mice on a Western diet display metabolic defects with age [18] to assess the impact of RSCE. All animals gained weight (approximately 15%) after the introduction of the Western diet and RSCE did not significantly alter the final body or white adipose tissue weight (Figure 2A,B,F). The number and area of white adipocytes were not significantly modified by the RSCE (Figure 2G–I). However, RSCE improved glucose tolerance as RSCE mice displayed a large decrease in blood glucose 30 min post-injection and a smaller area under the curve, compared to non-exposed 3xTg-AD mice (Figure 2C,D), without modification of fasting insulin (Figure 2E). Finally, circulating triglycerides levels were halved by both RSCE and acute cold exposure (4 °C, 24 h) (Figure 2J).

### 3.3 Repeated short cold exposures protect old 3xTg-AD mice against cold-induced tau phosphorylation

**Figure 3:**
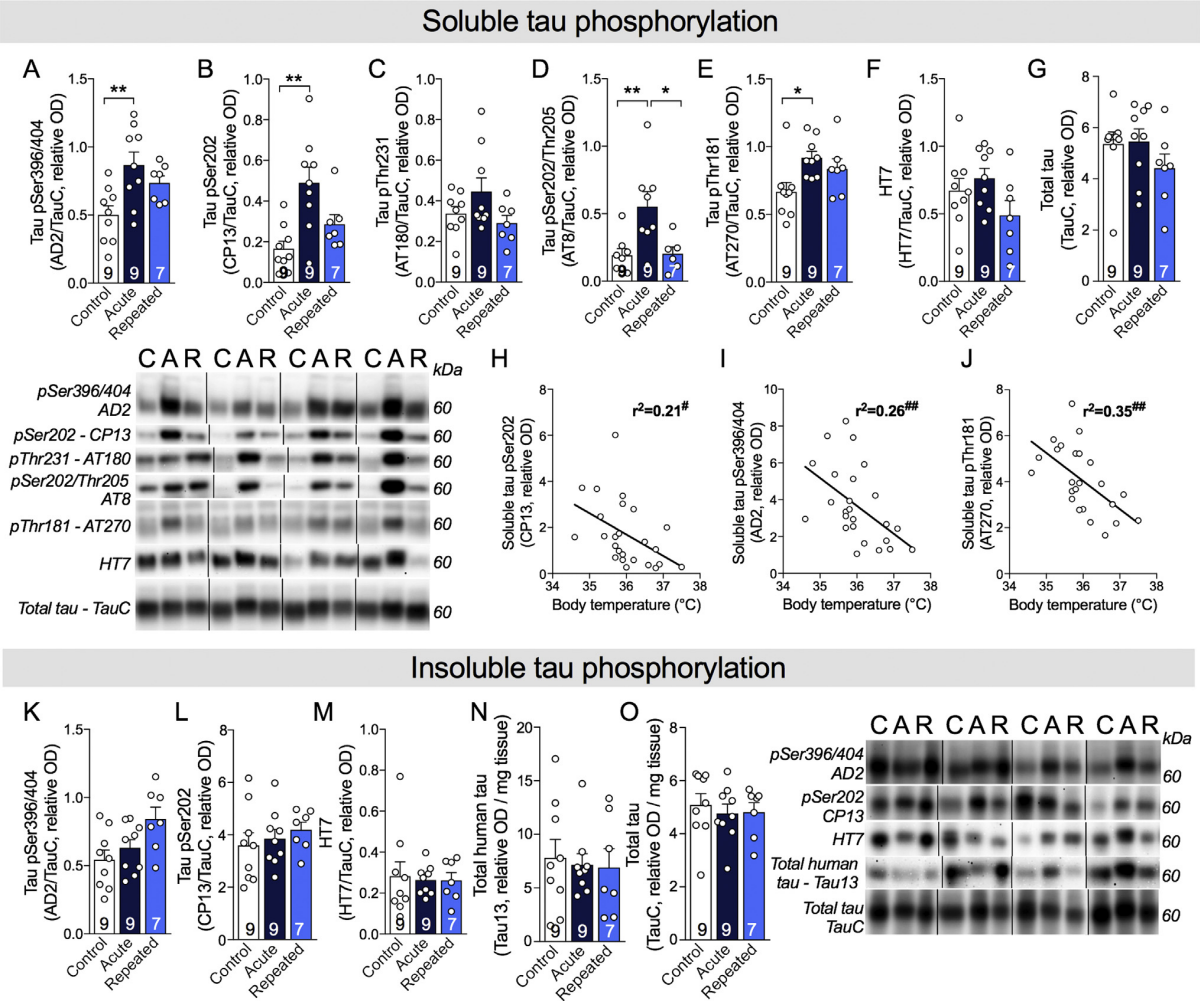
Repeated short cold exposures protect old 3xTg-AD mice against cold-induced phosphorylation of soluble tau. A–G: Hippocampal soluble tau phosphorylation of 16-month-old 3xTg-AD mice. H–J: Correlations between hippocampal soluble tau phosphorylation and body temperature at sacrifice. K–O: Hippocampal insoluble tau phosphorylation. All homogenates were run on the same gel, but consecutive bands were not taken for all representative photo examples. Data are represented as mean ± SEM (n/group indicated in bars). Statistical analyses: One-way ANOVA, Tukey's post-hoc test: *p < 0.05; **p < 0.01. Pearson r correlation: ^#^p < 0.05; ^##^p < 0.01. Control (C): 22 °C; Acute (A): 24 h, 4 °C; Repeated (R): 4 h, 4 °C for four weeks + 24 h, 4 °C.

Tau phosphorylation is one of the main neuropathological hallmarks of AD, strongly correlating with antemortem cognitive deficits [1], [2]. Cold-induced tau phosphorylation has been repeatedly observed in non-transgenic and in AD mouse models [8], [9], [10], [11], [12]. In this study, we first confirmed that acute exposure to cold (24 h, 4 °C) markedly increased the phosphorylation of soluble tau in the hippocampus of old 3xTg-AD mice (pSer394/404: +87%; pSer202: +211%; pSer202/Thr205: +183%; pThr181: +49% vs. non-exposed mice) (Figure 3A–G). As expected, tau phosphorylation strongly correlated with body temperature recorded at sacrifice (Figure 3H–J). Interestingly, RSCE completely prevented cold-induced tau hyperphosphorylation in the hippocampus, as the level of tau phosphorylation in RSCE mice was similar to non-exposed mice (Figure 3A–G). These results indicate that old 3xTg-AD mice undergoing BAT training with RSCE developed a sufficient thermogenic capacity to maintain their body temperature over a 24-hour cold challenge and thus preserved a level of tau phosphorylation similar to non-exposed mice. However, levels of insoluble tau in the hippocampus remained unchanged between exposed and non-exposed 3xTg-AD mice (Figure 3K–O).

**Figure 4:**
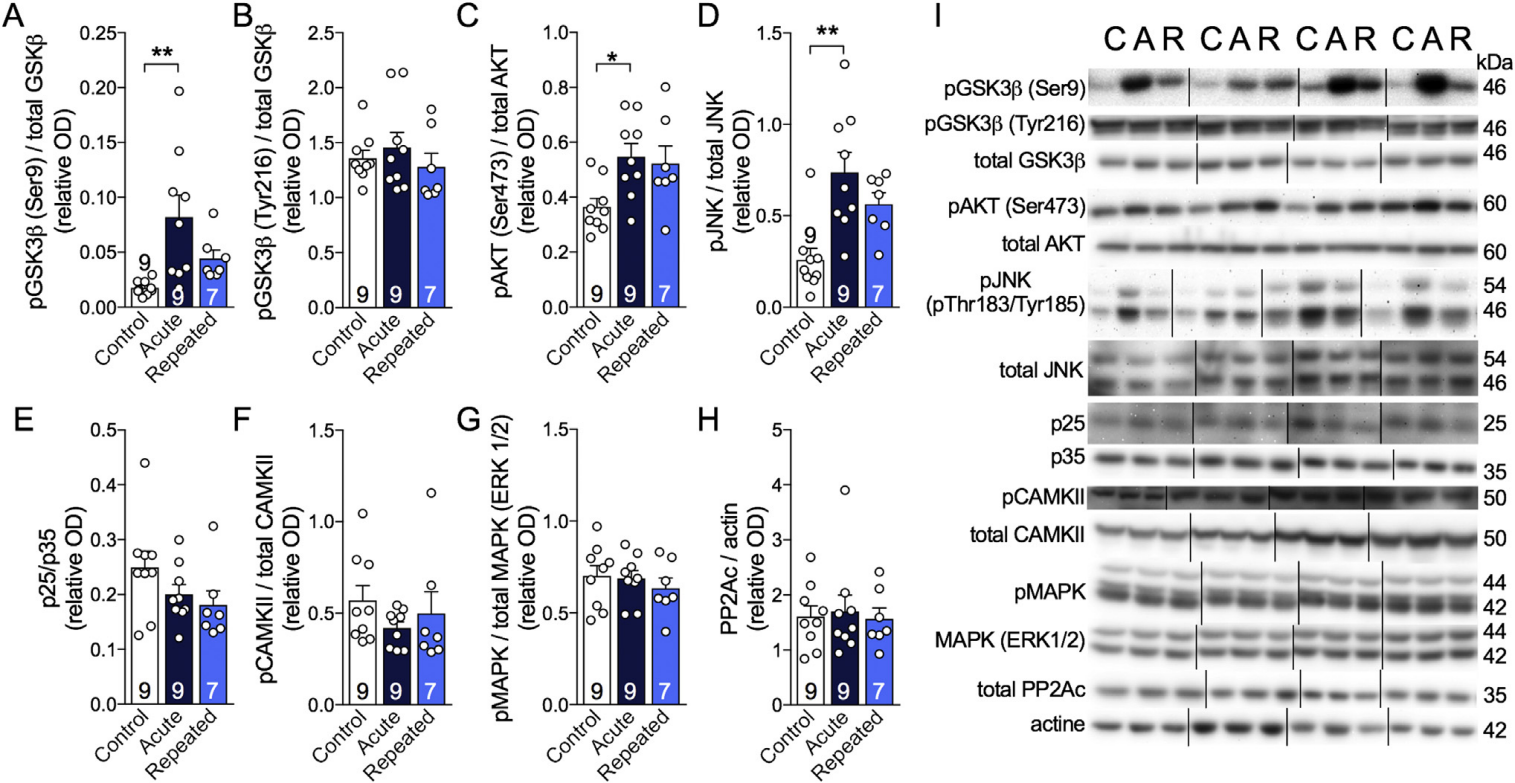
Repeated short cold exposures modify the levels of kinases involved in tau phosphorylation. A–I: Levels of kinases and phosphatase measured by western blot in the hippocampus of 16-month-old 3xTg-AD mice. All homogenates were run on the same gel, but consecutive bands were not taken for all representative photo examples. Data are represented as mean ± SEM (n/group indicated in bars). Statistical analyses: One-way ANOVA, Tukey's post-hoc test: *p < 0.05; **p < 0.01. Control (C): 22 °C; Acute (A): 24 h, 4 °C; Repeated (R): 4 h, 4 °C for four weeks + 24 h, 4 °C. Abbreviations: AKT: protein kinase B; CAMKII: calmodulin-dependent protein kinase II; GSK3β: glycogen synthase kinase 3β; JNK: c-Jun N-terminal kinase; MAPK (ERK1/2): mitogen-activated protein kinase; p25/35: protein kinase 25/35; PP2Ac: protein phosphatase 2A, catalytic subunit.

We then investigated the impact of RSCE on kinases and phosphatases involved in the phosphorylation of tau in the hippocampus (Figure 4A–I). We found that phospho-GSK3β (Ser9) increased after acute cold exposure (4 °C, 24 h) but remained equivalent to the control level after RSCE, thus following the same pattern as tau phosphorylation (Figure 4A). In contrast, phospho-GSK3β (Tyr216) levels remained similar in all groups (Figure 4B). Levels of phospho-JNK and phospho-AKT also increased following acute cold exposure, but with no significant effect from RSCE (Figure 4C,D). No significant changes were found for p25/p35, pCAMKII, pMAPK, and PP2Ac (Figure 4A–H).

Amyloid pathology accumulates progressively in the 3xTg-AD mice [42]. Here, RSCE and acute cold exposure (4 °C, 24 h) had no effect on either soluble or insoluble forms of Aβ40 or Aβ42 measured in the parieto-temporal cortex of the mice (Figure S3).

### 3.4 Repeated short cold exposures lead to FGF21 accumulation in plasma

**Figure 5:**
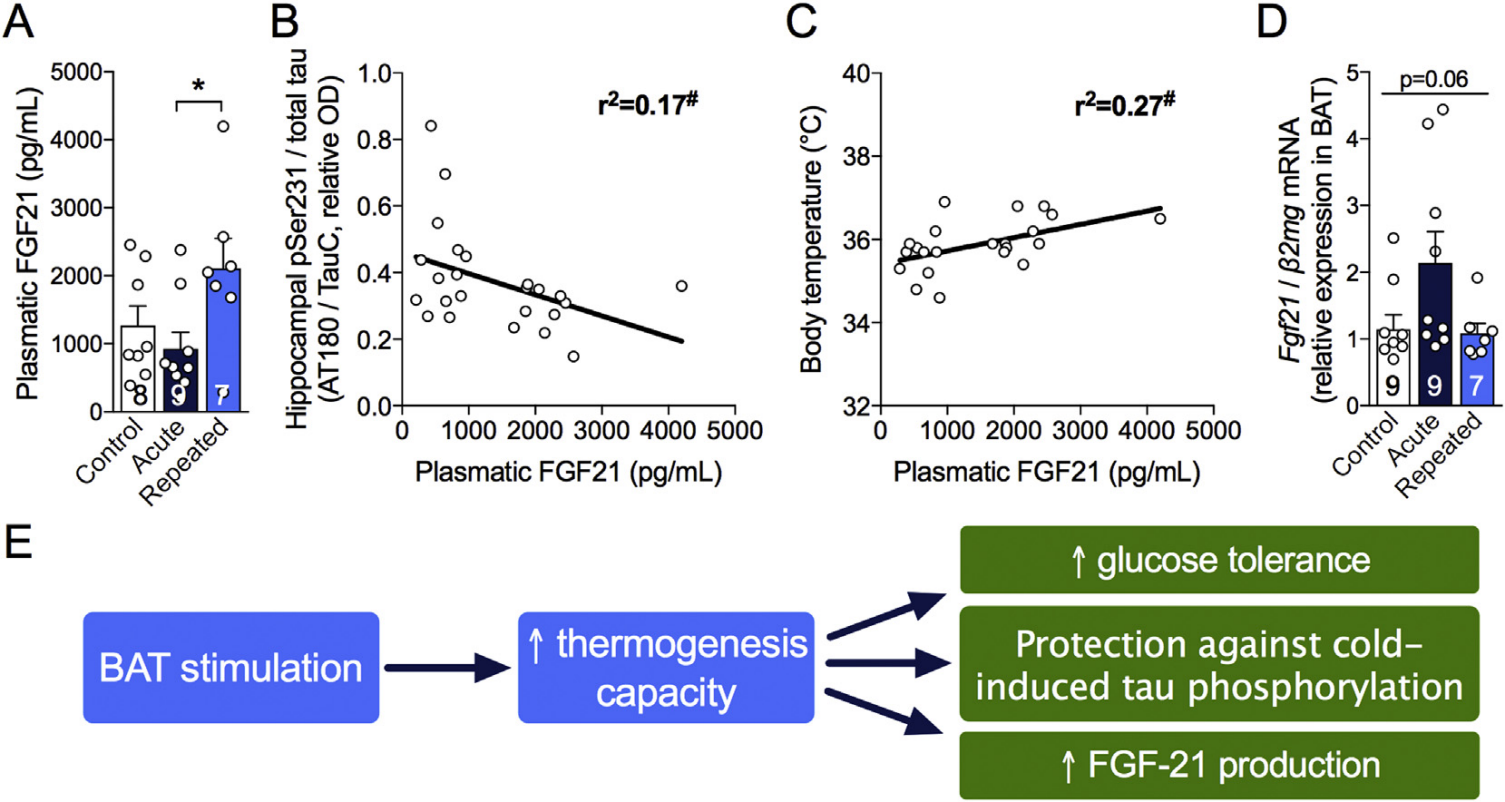
Repeated short cold exposures increase FGF21 production by BAT. A: FGF21 levels measured by ELISA in plasma sampled just before intracardiac perfusion in non-fasted mice. B,C: Correlations between plasma FGF21 and hippocampal tau phosphorylation at Ser231 and body temperature at sacrifice. D: Ratio of *Fgf21* on *β2mg* gene expression in BAT measured by qPCR. E: Schema of key results. Data are represented as mean ± SEM (n/group indicated in bars). Statistical analyses: One-way ANOVA, Tukey's post-test: *p < 0.05. Pearson r correlation: ^#^p < 0.05. Control: 22 °C; Acute: 24 h, 4 °C; Repeated; 4 h, 4 °C for four weeks + 24 h, 4 °C.

BAT has been recently presented as a secretory organ [30], communicating with other tissues, including the brain, through the secretion of batokines. Here, we focused on FGF21, because recent studies indicate that this hormone has neuroprotective properties [31], [33], [35]. We found that plasma levels of FGF21 increased by 66% following RSCE compared to control (Figure 5A). Surprisingly, plasma FGF21 concentrations were negatively correlated with levels of soluble tau pSer231 in the hippocampus of 3xTg-AD mice (Figure 5B), suggesting that FGF21 production induced by RSCE could be involved in the observed protection against cold-induced tau phosphorylation. In addition, plasma levels of FGF21 were correlated with body temperature recorded just before sacrifice (Figure 5C). Since BAT can secrete FGF21, we measured its expression in BAT of old 3xTg-AD mice and found that mice that only underwent the 24-hour cold challenge displayed a non-statistically significant trend toward higher *Fgf21* expression (Figure 5D).

## 4 Discussion

Based on previous work suggesting that thermoregulatory deficits contribute to AD pathogenesis [11], [12], we sought to determine whether stimulation of BAT thermogenesis counteracts metabolic and thermoregulation deficits as well as AD-like neuropathology in an animal model of AD. We opted for a non-pharmacological means to stimulate thermogenesis, namely RSCE, routinely used in the field of metabolic research, although not specific to BAT [36], [37], [38]. First, we found that RSCE was effective at increasing BAT thermogenic capacity and improving glucose tolerance in old 3xTg-AD mice. Notably, 3xTg-AD mice trained with RSCE were completely protected from cold-induced tau hyperphosphorylation, a key correlate of AD symptoms in patients. Finally, RSCE increased plasma FGF21 levels, a batokine involved in both metabolic improvement and neuroprotection [30], [31].

### 4.1 Increased thermogenesis capacity following RSCE in old mice

The present data show that RSCE increased BAT thermogenic capacity, as shown by increased BAT *Ucp1* expression and by the capacity of mice to resist cold-induced decreases in body temperature. WAT can also contribute to thermogenesis, although to a lower magnitude compared to interscapular BAT, by adopting a “browning” phenotype [36]. Here, we observed such a phenotype through the trend to increase in UCP1 staining in the WAT of repeatedly exposed mice. Since RSCE stimulates whole body thermogenesis and cold acclimation not only through BAT, we also measured markers of muscular thermogenesis but did not observe any modification in the mice repeatedly exposed to cold compared to control. RSCE or cold acclimation in humans have been shown to increase energy expenditure and BAT thermogenic activity [39], [40], [41]. However, studies in the elderly are lacking, possibly due to the risks associated with cold exposure in that population. Thermoregulation is impaired with aging, the BAT is less prominent in the elderly and old mice are more vulnerable to tau hyperphosphorylation induced by cold challenge compared to younger ones [11], [25]. Yet, our data confirm that RSCE is an appropriate research tool to investigate the effect of BAT stimulation even in old mice.

It should be noted that animals in our facilities are kept at 22 °C, which is lower than the thermoneutral temperature of the mouse estimated at 28 °C [62]. A previous study showed that thermoneutrality improves behavior and AD pathology in old 3xTg-AD mice after one week [12]. In addition, it is known that, when mice are housed at their thermoneutral temperature, their thermogenesis and sympathetic tone are halted while their adiposity is increased [50]. Therefore, the present results are valid in the context of mice experiencing mild thermogenesis and could be different at another environmental temperature.

### 4.2 RSCE improved peripheral metabolic disorders

Boosting thermogenic capacity could improve AD through different mechanisms. First, improvement in glucose and lipid metabolism, as well as insulin sensitivity, gained with higher thermogenesis may be therapeutic in AD. Type 2 diabetes and obesity are now widely listed among risk factors for developing AD, now increasingly recognized as a metabolic disease, since patients display impaired brain glucose metabolism and insulin signaling [14], [15], [16]. Peripheral metabolic impairments are also replicated in mouse models of AD neuropathology, which become aggravated following the induction of experimental diabetes [21], [51]. RSCE extended over three weeks (15 sessions of 4-hour cold exposure) was sufficient to improve glucose tolerance in 16-month-old 3xTg-AD mice. Such a result is in agreement with several studies that found that cold acclimation improved glucose metabolism in rodents and humans [38], [41]. Here, glucose intolerance in the 3xTg-AD mice was likely caused by AD neuropathology generated by the expression of transgenes in the CNS in combination with the Westernized diet [18], [21]. This indicates that BAT stimulation, a procedure with a long history in the field of metabolic diseases [24], could correct a peripheral metabolic endpoint consequent of central AD neuropathology.

### 4.3 RSCE protected against cold-induced tau phosphorylation

As a second key mechanism, BAT stimulation could exert a central therapeutic effect by preventing body temperature from dropping below the hypothermic threshold known to trigger tau hyperphosphorylation [11], [12]. Several studies in rodents have shown that tau phosphorylation is increased following a reduction in body temperature induced by different means, such as external cold exposure, anesthesia, hibernation, or glucose deprivation [8], [9], [10], [11], [12], [52]. Here, RSCE training before a 24-hour exposure to 4 °C completely blunted cold-induced tau phosphorylation in old 3xTg-AD mice. This indicates that an increased thermogenesis capacity confers full protection against this neuropathological insult. To the best of our knowledge, this is the first time that an intervention is shown to block an in vivo rise in cold-induced tau phosphorylation in the hippocampus, indicating an important link between thermoregulation and tau phosphorylation.

Although not all the studies found correlations between tau and cognition in humans, there is strong evidence that postmortem tau pathology correlates with antemortem memory deficits, and this association is usually stronger than with Aβ [1], [2], [53], [54], [55], [56]. The phospho-epitopes of tau protein assessed here, specifically pSer396/404 and pThr231, are important from an AD standpoint, as they correlate with clinical diagnoses and antemortem cognitive deficits, including episodic memory [1], [2]. Indeed, we previously observed a negative correlation between global cognitive score (including episodic memory) and both pThr231/Ser235 and pSer396/404 phosphorylated tau in the same series of clinically characterized volunteers from the Religious Order study, using extracts from the parietal cortex [2]. These associations remained significant even after adjustment for total tau, highlighting the importance of soluble tau phosphorylation at these sites in AD cognitive impairment [2]. In addition, the Ser396/404 epitope also correlates strongly with the spread of neurofibrillary tangles and Braak diagnosis [1]. In vitro evidence indicates that these phospho-epitopes are also involved in the pathological aggregation of tau [57]. Thus, it could be speculated that preventing hyperphosphorylation at those specific sites could prevent tau protein aggregation and its associated cognitive deficits.

Acute reductions in body temperature by various means, such as anesthesia, have been hypothesized to induce lasting deleterious rises in tau phosphorylation [8], [10]. Frequent and pronounced fluctuations in body temperature throughout the day, including hypothermia, are observed in the elderly, notably in AD patients [5], [6]. We previously showed that old age potentiates cold-induced tau phosphorylation in mice [11]. It is thus reasonable to think that tau phosphorylation fluctuates during the day along with body temperature, possibly leading to tau hyperphosphorylation and aggregation over the long term in older individuals. Here, RSCE over a four-week period did not increase insoluble tau phosphorylation, even if body temperature decreased during the first exposures (−0.5 to −1 °C). This indicates that short exposures to cold do not induce tau aggregation, and that the stimulation of thermogenesis may counteract the adverse impact of transient reductions in body temperature.

Tau phosphorylation is regulated by the activity of various kinases and phosphatases. Although we did not find any change in PP2A levels in the hippocampus of RSCE-exposed mice, PP2A activity has been shown to be directly inhibited by low temperatures [9]. Instead, GSK3β pSer9 followed a pattern very similar to tau phosphorylation, suggesting a possible involvement in the protection induced by RSCE. Such a view is consistent with the fact that this site of phosphorylation is sensitive to hypothermia [58].

### 4.4 RSCE increased FGF21 production

Our data suggest that FGF21 could be a third potential therapeutic target linking increased BAT thermogenesis activity with a potential benefit in AD. The idea that FGF21 is of interest in AD is in agreement with previous work suggesting that this batokine protects against obesity-induced memory impairment and Aβ42 toxicity [31], [35]. While the fact that RSCE mice were completely protected from cold-induced tau phosphorylation is certainly linked to the increased ability of old mice to maintain their body temperature during a cold challenge, a direct BAT-to-brain crosstalk could be at least partly involved. Plasma levels of FGF21 were inversely correlated with tau phosphorylation in the hippocampus of 3xTg-AD mice, which supports such a link but does not prove any causal relationship. Thus, further investigations on the effects of FGF21 in AD models, for example by directly administering recombinant FGF21 or drugs targeting the FGF21 receptor, would be of interest.

The present work does not confirm whether BAT training leads to improved cognitive faculties. The RSCE paradigm used here is not compatible with behavioral testing, as it is not possible to test the mice immediately after or during a cold exposure session. Indeed, locomotor activity and exploratory behavior are affected by a colder environment [59], [60]. Thus, mice will hardly perform as expected in most behavioral paradigms, introducing insurmountable biases. However, given the previously reported beneficial effects of RSCE on metabolic disorders, we opted to perform a glucose tolerance test during the few days without short cold exposure. Nevertheless, the effects observed in the present study following RSCE have been previously associated with cognitive-enhancing properties. Indeed, increased circulating FGF21 [31] and better glucose homeostasis [61] are associated with improved cognitive performance in diabetic rodents.

### 4.5 Conclusions

Overall, our results indicate for the first time that BAT thermogenesis stimulation through RSCE is efficient in old mice and results in improved glucose metabolism and protection against cold-induced tau hyperphosphorylation. Thus, therapeutic strategies aiming to stimulate BAT thermogenesis initially developed against metabolic disorders could be repurposed for dementia.

## Author contributions

M.T. developed the experimental design, performed animal experiments, protein extractions, western blots in the hippocampus, BAT and muscle, Aβ, insulin, and FGF21 ELISAs, qPCR, UCP1 immunostaining, and triglycerides assays, completed the statistical analysis, and wrote the first versions of the manuscript. P.B. contributed to animal experiments, protein extractions, western blots in the hippocampus and BAT, and Aβ and corticosterone ELISAs, and reviewed the manuscript. R.M. performed western blots in the hippocampus and contributed to the qPCR experiment and protein extractions. T.V. contributed to the UCP1 immunostaining, adipocytes quantification, and western blots in the muscle. E.P. provided expertise for the tau analysis and reviewed the manuscript. S.H. provided qPCR equipment and reviewed the manuscript. F.C. secured funding, contributed to the experimental design, and wrote the manuscript. F.C. is the guarantor of this work and, as such, had full access to all the data in the study and takes responsibility for the integrity of the data and the accuracy of the data analysis.

## Declarations of interest

None.

## Funding

M.T. was funded by a scholarship from the Alzheimer Society of Canada. P.B. received a scholarship from the Canadian Institutes of Health Research. This study was made possible by funding from the Canadian Institutes of Health Research (MOP 102532), the Quebec Network for Research on Aging, the Alzheimer Society of Canada (#1502), and the Canadian Foundation for Innovation (#34480). F.C. and E.P are a Fonds de Recherche du Québec - Santé (FRQ-S) scholars (#253895, #26936, and #252178).

## References

[1] Tremblay C., Pilote M., Phivilay A., Emond V., Bennett D.A., Calon F. (2007). Biochemical characterization of Abeta and tau pathologies in mild cognitive impairment and Alzheimer's disease. Journal of Alzheimer's Disease : JAD.

[2] Tremblay C., Francois A., Delay C., Freland L., Vandal M., Bennett (2017). Association of neuropathological markers in the parietal cortex with Antemortem cognitive function in persons with mild cognitive impairment and Alzheimer disease. Journal of Neuropathology and Experimental Neurology.

[3] Scheltens P., Blennow K., Breteler M.M.B., De Strooper B., Frisoni G.B., Salloway S. (2016). Alzheimer's disease. Lancet.

[4] Degroot D.W., Kenney W.L. (2007). Impaired defense of core temperature in aged humans during mild cold stress. AJP: Regulatory, Integrative and Comparative Physiology.

[5] Collins K. (1995). Hypothermia: the elderly person's enemy. The Practitioner.

[6] Gomolin I.H., Aung M.M., Wolf-Klein G., Auerbach C. (2005). Older is colder: temperature range and variation in older people. Journal of the American Geriatrics Society.

[7] Frisard M.I., Broussard A., Davies S.S., Roberts L.J., Rood J., de Jonge L. (2007). Aging, resting metabolic rate, and oxidative damage: results from the Louisiana Healthy Aging Study. The Journals of Gerontology. Series a, Biological Sciences and Medical Sciences.

[8] Planel E., Bretteville A., Liu L., Virag L., Du A.L., Yu W.H. (2009). Acceleration and persistence of neurofibrillary pathology in a mouse model of tauopathy following anesthesia. FASEB Journal : Official Publication of the Federation of American Societies for Experimental Biology.

[9] Planel E., Miyasaka T., Launey T., Chui D.-H., Tanemura K., Sato S. (2004). Alterations in glucose metabolism induce hypothermia leading to tau hyperphosphorylation through differential inhibition of kinase and phosphatase activities: implications for Alzheimer's disease. The Journal of Neuroscience.

[10] Le Freche H., Brouillette J., Fernandez-Gomez F.-J., Patin P., Caillierez R., Zommer N. (2012). Tau phosphorylation and sevoflurane anesthesia: an association to postoperative cognitive impairment. Anesthesiology.

[11] Tournissac M., Vandal M., Francois A., Planel E., Calon F. (2017). Old age potentiates cold-induced tau phosphorylation: linking thermoregulatory deficit with Alzheimer's disease. Neurobiology of Aging.

[12] Vandal M., White P.J., Tournissac M., Tremblay C., St-Amour I., Drouin-Ouellet J. (2016). Impaired thermoregulation and beneficial effects of thermoneutrality in the 3×Tg-AD model of Alzheimer's disease. Neurobiology of Aging.

[13] Baglietto-Vargas D., Shi J., Yaeger D.M., Ager R., LaFerla F.M. (2016). Diabetes and Alzheimer's disease crosstalk. Neuroscience and Biobehavioral Reviews.

[14] Chatterjee S., Peters S.A.E., Woodward M., Mejia Arango S., Batty G.D., Beckett N. (2016). Type 2 diabetes as a risk factor for dementia in women compared with men: a pooled analysis of 2.3 million people comprising more than 100,000 cases of dementia. Diabetes Care.

[15] An Y., Varma V.R., Varma S., Casanova R., Dammer E., Pletnikova O. (2018). Evidence for brain glucose dysregulation in Alzheimer's disease. Alzheimer's and Dementia: the Journal of the Alzheimer's Association.

[16] Arnold S.E., Arvanitakis Z., Macauley-Rambach S.L., Koenig A.M., Wang H.-Y., Ahima R.S. (2018). Brain insulin resistance in type 2 diabetes and Alzheimer disease: concepts and conundrums. Nature Reviews Neurology.

[17] (2016). Increased susceptibility to metabolic dysregulation in a mouse model of Alzheimer's disease is associated with impaired hypothalamic insulin signaling and elevated BCAA levels. Alzheimer's and Dementia.

[18] Vandal M., White P.J., Chevrier G., Tremblay C., St-Amour I., Planel E. (2015). Age-dependent impairment of glucose tolerance in the 3xTg-AD mouse model of Alzheimer's disease. FASEB Journal : Official Publication of the Federation of American Societies for Experimental Biology.

[19] El-Khoury N.B., Gratuze M., Petry F., Papon M.-A., Julien C., Marcouiller F. (2016). Hypothermia mediates age-dependent increase of tau phosphorylation in db/db mice. Neurobiology of Disease.

[20] Gratuze M., El-Khoury N.B., Turgeon A., Julien C., Marcouiller F., Morin F. (2017). Tau hyperphosphorylation in the brain of ob/ob mice is due to hypothermia: importance of thermoregulation in linking diabetes and Alzheimer's disease. Neurobiology of Disease.

[21] Vandal M., White P.J., Tremblay C., St-Amour I., Chevrier G., Emond V. (2014). Insulin reverses the high-fat diet-induced increase in brain aβ and improves memory in an animal model of Alzheimer disease. Diabetes.

[22] Yarchoan M., Arnold S.E. (2014). Repurposing diabetes drugs for brain insulin resistance in Alzheimer disease. Diabetes.

[23] Cannon B., Nedergaard J. (2004). Brown adipose tissue: function and physiological significance. Physiological Reviews.

[24] de Souza C.J., Burkey B.F. (2001). Beta 3-adrenoceptor agonists as anti-diabetic and anti-obesity drugs in humans. Current Pharmaceutical Design.

[25] Cypess A.M., Lehman S., Williams G., Tal I., Rodman D., Goldfine A.B. (2009). Identification and importance of brown adipose tissue in adult humans. The New England Journal of Medicine.

[26] Lee P., Greenfield J.R. (2015). Non-pharmacological and pharmacological strategies of brown adipose tissue recruitment in humans. Molecular and Cellular Endocrinology.

[27] Stanford K.I., Middelbeek R.J.W., Townsend K.L., An D., Nygaard E.B., Hitchcox K.M. (2013). Brown adipose tissue regulates glucose homeostasis and insulin sensitivity. The Journal of Clinical Investigation.

[28] Chondronikola M., Volpi E., Børsheim E., Porter C., Annamalai P., Enerbäck S. (2014). Brown adipose tissue improves whole-body glucose homeostasis and insulin sensitivity in humans. Diabetes.

[29] Ouellet V., Routhier-Labadie A., Bellemare W., Lakhal-Chaieb L., Turcotte E., Carpentier A.C. (2011). Outdoor temperature, age, sex, body mass index, and diabetic status determine the prevalence, mass, and glucose-uptake activity of 18F-FDG-detected BAT in humans. The Journal of Clinical Endocrinology and Metabolism.

[30] Villarroya F., Cereijo R., Villarroya J., Giralt M. (2017). Brown adipose tissue as a secretory organ. Nature Reviews Endocrinology.

[31] Sa-Nguanmoo P., Tanajak P., Kerdphoo S., Satjaritanun P., Wang X., Liang G. (2016). FGF21 improves cognition by restored synaptic plasticity, dendritic spine density, brain mitochondrial function and cell apoptosis in obese-insulin resistant male rats. Hormones and Behavior.

[32] Yu Z., Lin L., Jiang Y., Chin I., Wang X., Li X. (2018). Recombinant FGF21 protects against blood-brain barrier leakage through Nrf2 upregulation in type 2 diabetes mice. Molecular Neurobiology.

[33] Leng Y., Wang Z., Tsai L.-K., Leeds P., Fessler E.B., Wang J. (2015). FGF-21, a novel metabolic regulator, has a robust neuroprotective role and is markedly elevated in neurons by mood stabilizers. Molecular Psychiatry.

[34] Wang Z., Leng Y., Wang J., Liao H.-M., Bergman J., Leeds P. (2016). Tubastatin A, an HDAC6 inhibitor, alleviates stroke-induced brain infarction and functional deficits: potential roles of α-tubulin acetylation and FGF-21 up-regulation. Scientific Reports.

[35] Amiri M., Braidy N., Aminzadeh M. (2018). Protective effects of fibroblast growth factor 21 against amyloid-beta1-42-induced toxicity in SH-SY5Y cells. Neurotoxicity Research.

[36] Labbé S.M., Caron A., Chechi K., Laplante M., Lecomte R., Richard D. (2016). Metabolic activity of brown, “beige,” and white adipose tissues in response to chronic adrenergic stimulation in male mice. American Journal of Physiology. Endocrinology and Metabolism.

[37] Ravussin Y., Xiao C., Gavrilova O., Reitman M.L. (2014). Effect of intermittent cold exposure on brown fat activation. Obesity, and Energy Homeostasis in Mice.

[38] Wang T.-Y., Liu C., Wang A., Sun Q. (2015). Intermittent cold exposure improves glucose homeostasis associated with brown and white adipose tissues in mice. Life Sciences.

[39] Lee P., Smith S., Linderman J., Courville A.B., Brychta R.J., Dieckmann W. (2014). Temperature-acclimated brown adipose tissue modulates insulin sensitivity in humans. Diabetes.

[40] Blondin D.P., Labbé S.M., Tingelstad H.C., Noll C., Kunach M., Phoenix S. (2014). Increased brown adipose tissue oxidative capacity in cold-acclimated humans. The Journal of Clinical Endocrinology and Metabolism.

[41] Hanssen M.J.W., Hoeks J., Brans B., van der Lans A.A.J.J., Schaart G., van den Driessche J.J. (2015). Short-term cold acclimation improves insulin sensitivity in patients with type 2 diabetes mellitus. Nature Medicine.

[42] Oddo S., Caccamo A., Shepherd J.D., Murphy M.P., Golde T.E., Kayed R. (2003). Triple-transgenic model of Alzheimer's disease with plaques and tangles. Neuron.

[43] Lenhardt R. (2010). The effect of anesthesia on body temperature control. Frontiers in Bioscience (Scholar Edition).

[44] St-Amour I., Paré I., Tremblay C., Coulombe K., Bazin R., Calon F. (2014). IVIg protects the 3xTg-AD mouse model of Alzheimer's disease from memory deficit and Aβ pathology. Journal of Neuroinflammation.

[45] Petry F.R., Pelletier J., Bretteville A., Morin F., Calon F., Hébert S.S. (2014). Specificity of anti-tau antibodies when analyzing mice models of Alzheimer's disease. Problems and Solutions.

[46] Cheng A., Yang Y., Zhou Y., Maharana C., Lu D., Peng W. (2016). Mitochondrial SIRT3 mediates adaptive responses of neurons to exercise and metabolic and excitatory challenges. Cell Metabolism.

[47] Shi T., Wang F., Stieren E., Tong Q. (2005). SIRT3, a mitochondrial sirtuin deacetylase, regulates mitochondrial function and thermogenesis in brown adipocytes. The Journal of Biological Chemistry.

[48] Ahn B.-H., Kim H.-S., Song S., Lee I.H., Liu J., Vassilopoulos A. (2008). A role for the mitochondrial deacetylase Sirt3 in regulating energy homeostasis. Proceedings of the National Academy of Sciences of the United States of America.

[49] Bal N.C., Maurya S.K., Sopariwala D.H., Sahoo S.K., Gupta S.C., Shaikh S.A. (2012). Sarcolipin is a newly identified regulator of muscle-based thermogenesis in mammals. Nature Medicine.

[50] Cui X., Nguyen N.L.T., Zarebidaki E., Cao Q., Li F., Zha L. (2016). Thermoneutrality decreases thermogenic program and promotes adiposity in high-fat diet-fed mice. Physiological Reports.

[51] Gratuze M., Julien J., Petry F.R., Morin F., Planel E. (2017). Insulin deprivation induces PP2A inhibition and tau hyperphosphorylation in hTau mice, a model of Alzheimer's disease-like tau pathology. Scientific Reports.

[52] Arendt T., Stieler J., Strijkstra A.M., Hut R.A., Rüdiger J., Van der Zee E.A. (2003). Reversible paired helical filament-like phosphorylation of tau is an adaptive process associated with neuronal plasticity in hibernating animals. The Journal of Neuroscience.

[53] Arriagada P.V., Growdon J.H., Hedley-Whyte E.T., Hyman B.T. (1992). Neurofibrillary tangles but not senile plaques parallel duration and severity of Alzheimer's disease. Neurology.

[54] Nelson P.T., Alafuzoff I., Bigio E.H., Bouras C., Braak H., Cairns N.J. (2012). Correlation of Alzheimer disease neuropathologic changes with cognitive status: a review of the literature. Journal of Neuropathology and Experimental Neurology.

[55] Robinson J.L., Geser F., Corrada M.M., Berlau D.J., Arnold S.E., Lee V.M.Y. (2011). Neocortical and hippocampal amyloid-β and tau measures associate with dementia in the oldest-old. Brain : A Journal of Neurology.

[56] Bennett D.A., Schneider J.A., Wilson R.S., Bienias J.L., Arnold S.E. (2004). Neurofibrillary tangles mediate the association of amyloid load with clinical Alzheimer disease and level of cognitive function. Archives of Neurology.

[57] Martin L., Latypova X., Terro F. (2011). Post-translational modifications of tau protein: implications for Alzheimer's disease. Neurochemistry International.

[58] Bretteville A., Marcouiller F., Julien C., El-Khoury N.B., Petry F.R., Poitras I. (2012). Hypothermia-induced hyperphosphorylation: a new model to study tau kinase inhibitors. Nature Publishing Group.

[59] Ishii K., Kuwahara M., Tsubone H., Sugano S. (1996). The telemetric monitoring of heart rate, locomotor activity, and body temperature in mice and voles (Microtus arvalis) during ambient temperature changes. Laboratory Animals.

[60] Jhaveri K.A., Trammell R.A., Toth L.A. (2007). Effect of environmental temperature on sleep, locomotor activity, core body temperature and immune responses of C57BL/6J mice. Brain, Behavior, and Immunity.

[61] Porter D.W., Irwin N., Flatt P.R., Hölscher C., Gault V.A. (2011). Prolonged GIP receptor activation improves cognitive function, hippocampal synaptic plasticity and glucose homeostasis in high-fat fed mice. European Journal of Pharmacology.

[62] Cannon B., Nedergaard J. (2010). Nonshivering thermogenesis and its adequate measurement in metabolic studies. Journal of Experimental Biology.

