# Supplementary material for: Repeated cold exposures protect a mouse model of Alzheimer's disease against cold-induced tau phosphorylation
Source: Mol Metab. 2019 Jan 26;22:110–20. doi: 10.1016/j.molmet.2019.01.008 (PMC6437631; doi:10.1016/j.molmet.2019.01.008)
Supplement: Multimedia component 2 [file mmc2.docx]

**SUPPLEMENTAL MATERIAL**

**Table S1**. Composition of the “Western” diet.

AA, arachidonic acid; DHA, docosahexaenoic acid; DPA, docosapentaenoic acid; DTA, docosatetraenoic acid; EPA, eicosapentaenoic acid; LA, linoleic acid; LNA, linolenic acid; MUFA, monounsaturated fatty acid; OA, oleic acid; PUFA, polyunsaturated fatty acid; SFA, saturated fatty acid. †Measured by gas chromatography.

**Figure S1**. No difference between cold-exposed and non-exposed mice on change in plasma corticosterone levels.

Plasma corticosterone measured by ELISA in blood sampled before and after the first (A) and the thirteenth (B) 4-hour exposure at 4°C in non-fasted mice. All the animals were moved from their housing room (22°C) to the room next to the cold chamber to ensure equal stress between groups just before the exposure. Data are represented as mean ± SEM. n/group: 4-6. Statistical analyses: Paired t-test: # p<0.05 (before versus after); Unpaired Student t-test: not significant (ns, control versus repeated). Control: 22°C; Repeated; 4h, 4°C for four weeks + 24h, 4°C.

|  | **Control (n=9)** | | | **Acute (n=9)** | | | **Repeated (n=8)** | | | P-value |
| --- | --- | --- | --- | --- | --- | --- | --- | --- | --- | --- |
|  | Mean | ± | SD | Mean | ± | SD | Mean | ± | SD |  |
| **Sarcolipin** | 48,8 | ± | 18,2 | 53,4 | ± | 16,6 | 50,1 | ± | 12,0 | ns |
| **Serca1** | 77,9 | ± | 8,3 | 79,6 | ± | 11,0 | 81,3 | ± | 10,8 | ns |
| **Serca2** | 60,4 | ± | 13,0 | 62,4 | ± | 13,1 | 58,2 | ± | 7,6 | ns |
| **GLUT4** | 40,0 | ± | 11,9 | 50,6 | ± | 15,8 | 41,8 | ± | 16,9 | ns |
| **Pgc1-α** | 1,8 | ± | 1,1 | 2,2 | ± | 1,2 | 1,8 | ± | 0,8 | ns |

**Table S2**. Quantification of the optical density of proteins detected by western blot on gastrocnemius muscles homogenates.

Control: 22°C; Repeated; 4h, 4°C for four weeks + 24h, 4°C.

**Figure S2**. Change in body temperature before and after each short cold exposure (raw data of Fig.1B).

A: Description of the timeline for body temperature collection for each short cold exposure (4h, 4°C). B: Rectal temperatures of exposed (Repeated) and non-exposed (Control) mice taken before and after short exposures to cold (4h, 4°C) at 16 months. Paired t-test: ^@^p<0.05; ^@@^p<0.01.

**Figure S3**. Cortical levels of amyloid peptides are not affected by repeated short cold exposures.

Levels of soluble and insoluble Aβ40 and Aβ42 peptides measured by ELISA in the parieto-temporal cortex of 16-month-old 3xTg-AD mice. Data are represented as mean ± SEM. Control: 22°C; Repeated; 4h, 4°C for four weeks + 24h, 4°C.

|  | **Control** | | | **Acute** | | | **Repeated** | | |
| --- | --- | --- | --- | --- | --- | --- | --- | --- | --- |
|  | Mean |  | SD | Mean |  | SD | Mean |  | SD |
| **Synaptic proteins** |  |  |  |  |  |  |  |  |  |
| Drebrin | 2078 | ± | 938 | 2159 | ± | 887 | 1886 | ± | 627 |
| PSD95 | 4034 | ± | 1497 | 4017 | ± | 1352 | 4314 | ± | 773 |
| SNAP25 | 639 | ± | 236 | 747 | ± | 258 | 830 | ± | 157 |
| Synaptophysin | 3476 | ± | 1079 | 3687 | ± | 912 | 3640 | ± | 817 |
| **Others** |  |  |  |  |  |  |  |  |  |
| Bax / Bcl-2 | 1,034 | ± | 0,157 | 1,022 | ± | 0,112 | 0,964 | ± | 0,123 |
| GFAP | 5204 | ± | 872 | 5478 | ± | 1272 | 5151 | ± | 1189 |
| GLUT1 55 kDa | 3584 | ± | 1156 | 4411 | ± | 1273 | 4323 | ± | 1643 |
| GLUT1 45 kDa | 7806 | ± | 2350 | 8625 | ± | 2108 | 8755 | ± | 2743 |
| GLUT4 | 3156 | ± | 1313 | 3323 | ± | 1516 | 3300 | ± | 1381 |

**Table S3**. Synaptic proteins and other markers are not affected by repeated cold exposures.

Data are expressed as relative optical densities. Bax: Bcl-2 associated X protein; GFAP: glial fibrillary acidic protein; PSD95: postsynaptic density protein 95; SNAP25: synaptosomal-associated protein 25.

**Table S4**. Antibodies used in the study.
